# Prediction of Drug–Target Interaction Networks from the Integration of Protein Sequences and Drug Chemical Structures

**DOI:** 10.3390/molecules22071119

**Published:** 2017-07-05

**Authors:** Fan-Rong Meng, Zhu-Hong You, Xing Chen, Yong Zhou, Ji-Yong An

**Affiliations:** 1 School of Computer Science and Technology, China University of Mining and Technology, Xuzhou 21116, China; mengfr@cumt.edu.cn (F.-R.M.); yzhou@cumt.edu.cn (Y.Z.); ajy@cumt.edu.cn (J.-Y.A.); 2 Xinjiang Technical Institute of Physics and Chemistry, Chinese Academy of Science, Urumqi 830011, China; 3 School of Information and Control Engineering, China University of Mining and Technology, Xuzhou 21116, China

**Keywords:** DTI, RVM, BIGP, PCA

## Abstract

Knowledge of drug–target interaction (DTI) plays an important role in discovering new drug candidates. Unfortunately, there are unavoidable shortcomings; including the time-consuming and expensive nature of the experimental method to predict DTI. Therefore, it motivates us to develop an effective computational method to predict DTI based on protein sequence. In the paper, we proposed a novel computational approach based on protein sequence, namely PDTPS (Predicting Drug Targets with Protein Sequence) to predict DTI. The PDTPS method combines Bi-gram probabilities (BIGP), Position Specific Scoring Matrix (PSSM), and Principal Component Analysis (PCA) with Relevance Vector Machine (RVM). In order to evaluate the prediction capacity of the PDTPS, the experiment was carried out on enzyme, ion channel, GPCR, and nuclear receptor datasets by using five-fold cross-validation tests. The proposed PDTPS method achieved average accuracy of 97.73%, 93.12%, 86.78%, and 87.78% on enzyme, ion channel, GPCR and nuclear receptor datasets, respectively. The experimental results showed that our method has good prediction performance. Furthermore, in order to further evaluate the prediction performance of the proposed PDTPS method, we compared it with the state-of-the-art support vector machine (SVM) classifier on enzyme and ion channel datasets, and other exiting methods on four datasets. The promising comparison results further demonstrate that the efficiency and robust of the proposed PDTPS method. This makes it a useful tool and suitable for predicting DTI, as well as other bioinformatics tasks.

## 1. Introduction

The identification of drug–target interactions (DTI) has recently emerged as an area of intense research activity due to its important role in finding new proteins to target for drug development and discovering new drug candidates [1,2]. However, the target proteins of many drugs are not complete or even not known. In the past years, much effort has been devoted to using experimental methods to identify drug–protein interactions. But these experimental methods are both time-consuming and expensive. It often costs billions of dollars for developing a successful novel chemistry-based drug and takes nearly a decade for introducing the drug to market. However, there are only few drug candidates that can be approved to reach the market by Food and Drug Administration (FDA) [3,4,5]. This is partially caused by the unacceptable toxicity for those drug candidates with the satisfactory activity, due to the deficient of the knowledge of drug–target interactions. Thus, it is necessary to develop fast and reliable computational methods for identifying drug–target interactions. Therefore, it is becoming more and more important to use computational approaches to detect DTI. The cost and time of experimental methods can be reduced and new potential drug–target interaction candidates can be found by using computational methods. 

With the emergence of molecular medicine and the completion of the human genome project, the body of publicly-available knowledge of biology and chemistry is increasing rapidly. It makes the researchers restudy DTI questions by a systematic integration. A number of related databases that focus on drug–target relations have been constructed. We can freely obtain some of them from the public sector, such as SuperTarget and Matador [6], Kyoto Encyclopedia of Genes and Genomes (KEGG) [7], DrugBank [8,9], Therapeutic Target Database (TTD) [10,11], etc. It is much useful for many researchers that a number of important experimental materials can be obtained from these databases to develop new computational approaches for identifying DTI on a genome-wide scale [12,13].

All the time, in order to predict drug–target interactions, traditional computational methods are divided into the ligand-based virtual screening method and the docking approach. The ligand-based virtual screening method compares the similarity of a given proteins represented based on chemical structure with a classic SAR framework, which is used to predict DTI [14]. However, there is an obvious shortcoming that the information of protein domains is not used for the method. The docking simulation is a much useful molecular modeling method that can detect the positive interactions by using dynamic simulation when drug molecule and protein bound to each other [15,16,17]. However, the method has also a significant disadvantage that it can be only applied to proteins whose 3D structures are known. However, up to now, the proteins whose 3D structures are known comprise only a small part of all proteins. As a result, it is difficult to satisfy the experimental condition of the docking simulation method. Furthermore, the number of detected protein sequence data related to the known 3D structure data are increasing exponentially. Therefore, this promotes the need for developing new computational approaches based on protein sequence for detecting drug–target interactions.

In recent years, a number of computational approaches have been proposed to predict drug–target interactions. For example, Yang et al. [18] developed a new computational method to detect multiple target optimal intervention solutions in a disease network. The method attempts to identify effective points of intervention and the combination of interventions within a given disease network, which can best restore the disease network to a desired normal state. Yan et al. [19] developed a representation of drug–target pairs based on drug chemical similarity and target sequence similarity and employed the random forest as classifier to build the prediction models. By comparing the method and the state-of-the-art methods, it produces satisfying performance on the benchmark datasets. Kuang et al. [20] developed a novel method that proposed an eigenvalue transformation technique and applied this technique to two representative algorithms for predicting DTI, the Regularized Least Squares classifier (RLS) and the semi-supervised link prediction classifier (SLP). The prediction results show that the method achieved better performance on drug–target interaction prediction. Bharadwaja et al. [21] proposed a new approach for identifying novel interactions for drugs and targets with no prior interaction information, which improved a machine learning method by integrating more correlated information of the drug compounds and extended it to a weighted profile method. Peng et al. [22] proposed a prediction model name as NormMulInf which is a semi-supervised-based learning framework through collaborative filtering theory, employing labeled and unlabeled interaction information. Firstly, the method determines similarity principles, for example samples’ similarities and local correlations between samples’ labels by integrating biological information. Secondly, the similarity information can be integrated into the NormMulInf model, which solves the problem of augmented Lagrange multipliers. Wang et al. [23] proposed a new computational method, namely PDTD (Predicting Drug Targets with Domains), for identifying potential target proteins of new drugs based on derived interactions between drugs and protein domains. Zhang et al. [24] proposed a stacking-based ensemble learning method to boost performance of previous DTI prediction methods by using a state-of-the-art support vector machine (SVM) model as classifier to integrate the prediction results of previous methods. Although these methods have achieved good prediction accuracy, however, the proposed prediction model focuses on improving the prediction accuracy. Thus, there is still room to improve the prediction accuracy to identify DTI.

**Figure 1:**
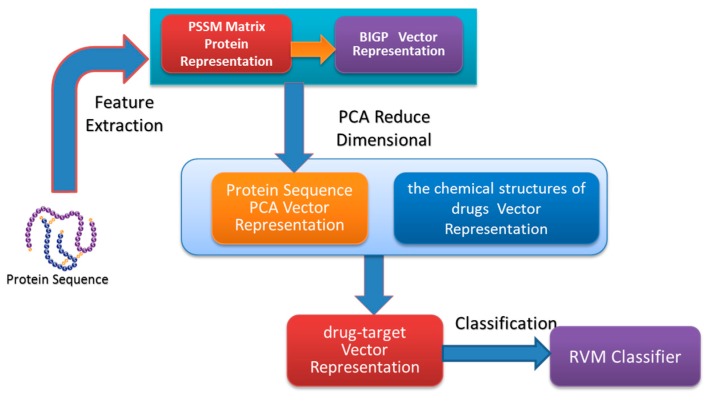
The flow chart of the proposed prediction model.

In the paper, we proposed a novel computational approach based on protein sequence, namely PDTPS (Predicting Drug Targets with Protein Sequence), to predict drug–target interactions (DTI). The PDTPS method combines Bi-gram probabilities (BIGP), Position Specific Scoring Matrix (PSSM), and Principal Component Analysis (PCA) with Relevance Vector Machine (RVM). In order to evaluate the prediction capacity of the PDTPS, we carry out the experiment on enzyme, ion channel, GPCR, and nuclear receptor datasets by using five-fold cross-validation tests. The proposed PDTPS method achieved average accuracy of 97.73%, 93.12%, 86.78%, and 87.78% on enzyme, ion channel, GPCR, and nuclear receptor datasets respectively. The experimental results showed that our method has good prediction performance. Furthermore, in order to further evaluate the prediction performance of the proposed PDTPS method, we compared it with the state-of-the-art support vector machine (SVM) classifier on enzyme and ion channel datasets and other exiting methods on four datasets. The promising comparison results further demonstrate the efficiency and robustness of the proposed PDTPS method. This makes it a useful tool and suitable for predicting DTI, as well as other bioinformatics tasks. The flow chart of the proposed prediction model is shown in Figure 1.

## 2. Results and Discussion

### 2.1. Performance of the Proposed Method

In order to verify the effectiveness of the proposed method, we carry out the experiment on enzyme, ion channel, GPCR, and nuclear receptor datasets through employing five-fold cross-validation tests respectively. For five-fold cross-validation, the whole dataset was divided into five parts; four parts of them were used as training samples, and one part of them was employed as testing samples. In addition, there are several parameters that need be optimized for the RVM classifier in the experiment. Here, the ’ploy2’ function was selected as the kernel function, we also set up other parameters: width = 1, initapla = 1/N and beta = 0. Where width represents the width of ‘ploy2’ kernel function, N is the number of training samples, and beta represents classification. Table 1, Table 2, Table 3 and Table 4 list the five-fold cross-validation tests prediction results by using the proposed approach on enzyme, ion channel, GPCR, and nuclear receptor datasets.

It can be observed from Table 1, Table 2, Table 3 and Table 4 that the average Accuracy (Ac) and its standard deviation for enzymes, ion channels, GPCRs, and nuclear receptors is 97.73%, 93.12%, 86.77%, 87.78%, and 0.40%, 1.34%, 2.41%, and 3.17%, respectively. The corresponding average Sensitivity (Sn) and its standard deviation is 97.44%, 93.32%, 84.89%, 92.63%, and 1.04%, 1.54%, 4.04%, 11.53%, respectively. The corresponding average Precision (Pe) and its standard deviation is 98.01%, 92.96%, 87.91%, 85.19%, and 0.78%, 2.10%, 3.47%, 6.70%, respectively. At the same time, the average Matthews’s correlation coefficient (Mcc) and its standard deviation is 95.56%, 87.18%, 76.97%, 78.32%, and 0.76%, 2.28%, 3.64%, 4.72%, respectively. These experimental results indicated that the proposed method can obtain good prediction accuracy for predicting drug–target interactions.

The good prediction results of the proposed approach for drug–target interactions result from the correct choice of feature extraction method and classifier. Major improvements of the proposed feature extraction method can be divided into three following reasons: (1) Because PSSM not only describes the order information but also retains sufficient prior information, it can capture useful information from a given protein sequence; (2) The Bi-gram probabilities represented each protein PSSM and calculated the Bi-gram feature through employing the probability information PSSM contains. Because the Bi-gram features extracted from PSSMs can significantly reduce the sparsity level, this helps in improving the recognition performance; (3) For reducing the influence of noise for classifying and ensuring the integrity of feature information, we transformed the dimensions of each BIGP feature vector from 400 to 350 using Principal Component Analysis (PCA). Thus, it can be seen from these experimental results that the proposed BIGP method plays an essential role for improving prediction accuracy for predicting DTI.

### 2.2. Comparison with the SVM-Based Method

The proposed method has achieved good prediction accuracy. In order to further evaluate the prediction performance of the RVM classifier, the comparison of prediction accuracy between the RVM classifier and the state-of-the-art support vector machine (SVM) classifier was carried out through employing the same feature extraction method on enzyme and ion channel datasets. We also adopted five-fold cross-validation tests to assess the prediction accuracy of the SVM classifier. The LIBSVM tool [25] of SVM was used to execute classification. In the experiment, we also optimized several parameters of the SVM classifier. We selected the radial basis function (RBF) as the kernel function, and the c and g parameters of the RBF kernel were set up (c = 0.5 and g = 0.6) by using a grid search method.

**Figure 2:**
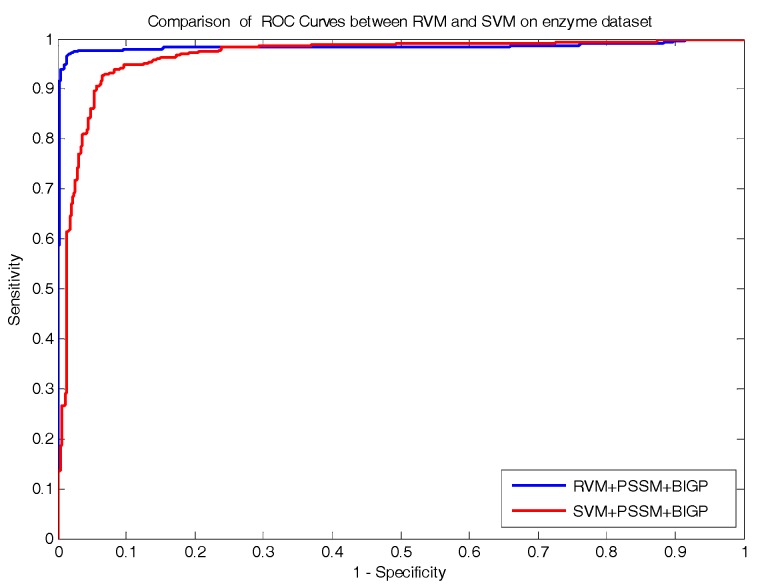
Comparison of ROC curves performed between RVM and SVM on an enzyme dataset.

**Figure 3:**
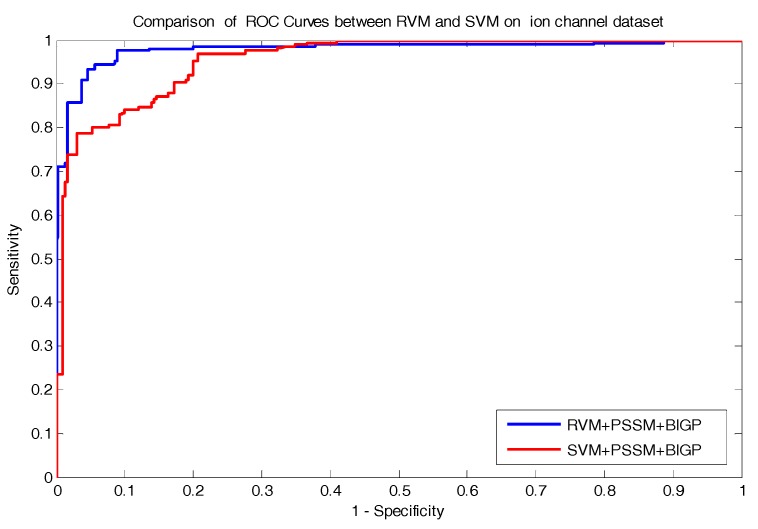
Comparison of ROC curves performed between RVM and SVM on an ion channel dataset.

The comparison prediction results of RVM and SVM classifiers on enzyme and ion channel datasets are listed in Table 5 and Table 6, respectively. At the same time, the comparison of ROC Curves between RVM and SVM classifiers are also shown in Figure 2 and Figure 3 on enzyme and ion channel datasets, respectively. As displayed in Table 5, the RVM classifier obtained 97.73% average accuracy on the enzyme dataset, while 91.15% average accuracy was achieved by the SVM classifier. Similarly, it can be seen form Table 6 that 93.12% average accuracy was obtained by the RVM classifier and 87.77% average accuracy was achieved by the SVM classifier on the ion channel dataset. It can be observed from these results that the prediction accuracy obtained by the RVM classifier is significantly higher than that of the SVM classifier. In addition, as displayed in Figure 2 and Figure 3, the ROC curves of the RVM classifier is also obviously better than that of the SVM classifier. The proposed method obtained good prediction results which may be attributable to two reasons: (1) because the RVM classifier greatly reduces the amount of calculation of the kernel function relative to the SVM classifier; which helps in improving the prediction performance; (2) the kernel functions required to meet the condition of Mercer is the obvious disadvantage of the SVM classifier; however, the RVM classifier overcame it and solved the problem. Thus, all of these experimental results indicate that the proposed prediction model might become a useful tool for predicting DTI, as well as performing other bioinformatics tasks.

### 2.3. Comparison with Other Methods

Up to now, a number of computational methods have been proposed for predicting drug target interactions. In our study, in order to further evaluate the prediction performance of the proposed method, we compared its prediction accuracy with four existing DTI predictors; DBSI [26], Yamanishi [27], KBMF2K [28], and NetCMP [29] on enzyme, ion channel, GPCR, and nuclear receptor datasets, respectively. These methods use the same strategy as the proposed method, however, they adopt different feature extraction methods and classifiers. Table 7 displays these comparison results. It can be observed from Table 7 that the prediction accuracy of the proposed approach is significantly higher than the other four methods on enzyme, ion channel, GPCR, and nuclear receptor datasets. The comparison results further demonstrated that the PDTPS can improve the prediction accuracy relative to current approaches. Due to using a good classifier and a novel feature extraction method, the proposed method achieved good prediction results. This makes the PDTPS a useful tool and suitable for predicting DTI.

## 3. Materials and Methods

### 3.1. Dataset

In this study, we carried out the experiment using the proposed method on four protein targets datasets: enzymes, ion channels, GPCRs, and nuclear receptors. These data can be freely obtained from the KEGG BRITE [7], BRENDA [30], SuperTarget [6], and Drug Bank [8] databases and were used as the gold-standard datasets by Yamanishi et al [27] The number of drugs known to target enzymes, ion channels, GPCRs, and nuclear receptors are 445, 210, 233, and 54, respectively. The numbers of proteins known to be targeted by the drugs are 664, 204, 95, and 26 respectively. These drug–target pairs were carefully screened, 5127 pairs of them are known to interact with each other. The numbers of known interactions involving enzymes, ion channels, GPCRs, and nuclear receptors are 2926, 1476, 635, and 90, respectively. Then, all known interactions of the drug–target pairs were chosen as positive sample sets for four datasets in our experiment.

A bipartite graph is usually used to represent a drug–target interaction network, whose nodes represent target proteins or drug molecules and the edges describe the real drug–target interactions that have been already identified through experiments or other ways. It can be observed from bipartite graph that the number of the real drug–target interactions edges are small. Here, we take the enzyme dataset as an example; there are a total of 295,480 (445 × 664) connections in the corresponding bipartite and only 2926 edges of them are known drug–target interactions. Therefore, the possible number of negative samples (295,480 − 2926 = 29,2554) is significantly more than the number of positive samples (2926), which is a bias problem. In order to solve this problem, we randomly selected the negative samples as much as the positive sample. As a result, there are 2926, 1476, 635, and 90 negative samples of enzymes, ion channels, GPCRs, and nuclear receptors datasets. In other words, there are 5852, 2952, 1270, and 180 drug–target pairs of enzymes, ion channels, GPCRs, and nuclear receptors datasets in the experiment.

### 3.2. Position Specific Scoring Matrix

Position Specific Scoring Matrix (PSSM) can be represented an M × 20 matrix $M\;=\;\left\{M_{ij}\;i:1\;=\;1\ldots M,j\;=\;1\ldots 20\right\}\;$, where M represents the length of a given protein sequence, 20 is the number of 20 amino acids, and $M_{ij}$ represents the score of the $j_{th}$ amino acid relative to the $i_{th}$ position for a query protein sequence [31]. The score $M_{ij}$ can be expressed as $M_{ij}\;=\;\overset{20}{\underset{k\;=\;1}{\sum }}p\left(i,k\right)\;\times \;q\left(j,k\right)$, where p(i,k) represents the appearing frequency of the $k_{th}$ amino acid at position i of the probe, and q(i,k) is the value of Dayhoff’s mutation matrix between $j_{th}$ and $k_{th}$ amino acids. Thus, a high score represents a highly-conserved position; on the contrary, a low score represents a weakly-conserved position.

In the study, in order to create experimental datasets, we used Position Specific Iterated BLAST (PSI-BLAST) [32] to construct PSSMs for each protein sequence. The e-value and number of iterations are set up as the default values in PSI-BLAST. For achieving highly and widely homologous sequences, an e-value of 0.001 and three iterations were selected. It is possible that features may be different if we use different parameters, however, in the work we concentrated on exploring general PSSM features for predicting DTI by employing mostly default settings. Thus, each PSSMs feature vector can be represented as M × 20 matrix by using PSI-BLAST, where M is the number of residues of a given protein sequence and the 20 columns are the number of 20 amino acids. 

### 3.3. Bi-Gram Probabilities

The Bi-gram Probabilities (BIGP) have been used for protein fold recognition. In the literature [33], it was described how to use a given protein’s original primary sequence or its consensus sequence for protein fold recognition. Instead, we employed the BIGP feature extraction method that the literature [34] proposed to represent a given protein sequence based on its PSSM (PSSM has been mentioned in the Section 3.2 of the paper). In detail, the bi-gram feature vector was computed through counting the bi-gram frequencies of occurrence in PSSM. It is assumed that *P* represents the PSSM of a protein sequence, which contains *L* rows and 20 columns, where *L* is the length of a given protein sequence and 20 columns represents a number of 20 amino acids. The PSSM element $P_{ij}$ can be interpreted as the relative probability of $j_{th}$ amino acid at the $i_{th}$ location of the primary protein sequence, $P_{ij}$ can be expressed as $P_{ij}\;=\;\overset{20}{\underset{j\;=\;1}{\sum }}i:1\;=\;1\ldots L,j\;=\;1\ldots 20$. The frequency of occurrence of transition from $m_{th}$ amino acid to $n_{th}$ amino acid can be defined as follows:

(1) $$BIGP_{mn}\;=\;\overset{L-1}{\underset{i\;=\;1}{\sum }}P_{i,m}P_{i+1,n}1\leq \;m\leq 20,\;1\leq n\leq 20$$

Equation (1) gives 400 frequencies of occurrence $BIGP_{mn}$ for 400 bi-gram transitions, the matrix BIGP called the bi-gram occurrence matrix, the number of the 400 whose elements represent the bi-gram feature vector [34] are as follows:

(2) $$BF\;=\;\left[BGP_{1,1},BGP_{1,2}\ldots BGP_{1,20},BGP_{2,1},\ldots BGP_{2,20},\ldots \ldots BGP_{20,1},\ldots BGP_{20,20}\right]$$

These bi-gram features can also be expressed as follows:

(3) $$BF\;=\;\left[\varphi_{1,},\varphi_{2},\varphi_{3,}\ldots \varphi_{u,},\ldots \varphi_{\theta }\right]$$

where θ = mn = 400 is the dimensionality of the feature vector BF, the $\varphi_{u}$ can be represented as follows:

(4) $$\varphi_{u}\;=\;\{\begin{array}{c} BGP_{1,u}\;\left(1\leq u\leq 20\right)\\ BGP_{2,u-20\;}\;\left(21\leq u\leq 40\right)\\ \ldots \ldots \;\\ BGP_{20,u-380\;}\;\left(381\leq u\leq 400\right)\\ \; \end{array}$$

Finally, each protein sequence was converted into a 400-dimensional vector by using BIGP method. In the paper, to reduce the influence of noise and improve the prediction accuracy, the dimensions of enzymes, ion channels, GPCRs, and nuclear receptors datasets were reduced from 400 to 350 by using Principal Component Analysis (PCA) method.

### 3.4. Relevance Vector Machine 

The related theory of the Relevance Vector Machine describes in details in the literature [35]. We assumed $\;{\left\{x_{n},t_{n}\right\}}_{n\;=\;1}^{N}$, $x_{n}\in R^{d}$ is the training set for binary classification question, where $t_{n}\in \left\{0,1\right\}$ represents the training set label, $t_{i}$ is the testing set label, and $t_{i}\;=\;y_{i}+\varepsilon_{i}$, where $y_{i}\;=\;w^{T}\varphi \left(x_{i}\right)\;=\;\overset{N}{\underset{j\;=\;1}{\sum }}w_{j}K\left(x_{i},x_{j}\right)+w_{0}$ is the classification model; $\varepsilon_{i}$ is the additional noise, with a mean value of zero and a variance of *σ*^2^, where $\varepsilon_{i}\sim N\left(0,\sigma^{2}\right),t_{i}\sim N\left(y_{i},\sigma^{2}\right).$ It is assumed that the training sets are independent and identically distributed; the vector *t* submits to as follows distribution:

(5) $$p\left(t|x,w,\sigma^{2}\right)={\left(2\pi \sigma^{2}\right)}^{-N/2}\exp[-\frac{1}{2\sigma^{2}}{\left|\left|t-\varphi w\right|\right|}^{2}]$$

where φ is defined as follows:

(6) $$\varphi =\left(\begin{array}{ccc} 1 & k\left(x_{1},x_{1}\right)\cdots & k\left(x_{1},x_{N}\right)\\ \ldots & \ldots & \ldots \\ 1 & k\left(x_{N},x_{1}\right)\ldots & k\left(x_{N},x_{N}\right) \end{array}\right)$$

The training set label *t* is employed to detect the testing set label $t_{*}$, given by

(7) $$p\left(t_{*}|t\right)=\int^{\text{​}}p\left(t_{*}|w,\sigma^{2}\right)p(w,\sigma^{2}|t)dwd\sigma^{2}$$

Due to making the value of most components of the weight vector w zero and reducing the number of calculation of the kernel function, additional conditions are attached to the weight vector w Assuming that $w_{i}$ obeys a distribution with a mean value of zero and a variance of $\alpha_{i}^{-1}$, the mean $w_{i}\sim N\left(0,\;\alpha_{i}^{-1}\right)$,$p\left(w|a\right)=\overset{N}{\underset{i=0}{\prod }}p(w_{i}|a_{i})$ where *α* is a hyper-parameter vector of the prior distribution of the weight vector w.

(8) $$p\left(t_{*}|t\right)=\int^{\text{​}}p\left(t_{*}|w,a,\sigma^{2}\right)p(w,a,\sigma^{2}{|t)\mathrm{dwdad}\sigma }^{2}$$

(9) $$p\left(t_{*}|w,a,\sigma^{2}\right)=N(t_{*}\;|y\left(x_{*};w\right),\sigma^{2})$$

Because $p\left(w,a,\sigma^{2}|t\right)$ cannot be obtained by an integral, it must be resolved using a Bayesian formula, given as

(10) $$p\left(w,a,\sigma^{2}|t\right)=p\left(w|a,\sigma^{2},t\right)p(a,\sigma^{2}|t)$$

(11) $$p\left(w|a,\sigma^{2},t\right)=p\left(t|w,\sigma^{2}\right)p\left(w|a\right)/p\left(t|a,\sigma^{2}\right)$$

The integral of the product of $p\left(w,a,\sigma^{2}|t\right)$ and p(w|a) is as follows:

(12) $$p\left(t|a,\sigma^{2}\right)={\left(2\pi \right)}^{-N/2}{\left|\Omega \right|}^{-1/2}\exp\left(-\frac{t^{T}\Omega^{-1}t}{2}\right)$$

(13) $$\Omega =\sigma^{2}I+\varphi A^{-1}\varphi^{T},\;A=diag\left(a_{0,}a_{1,}\ldots ,a_{N}\right)$$

(14) $$p\left(w|a,\sigma^{2},t\right)={\left(2\pi \right)}^{-\left(N+1\right)/2}{\left|\Sigma \right|}^{-1/2}\exp\left(-\frac{{\left(w-u\right)}^{T}\left(w-u\right)}{2}\right)$$

(15) $$\Sigma ={\left(\sigma^{-2}\varphi^{T}\varphi +A\right)}^{-1}$$

(16) $$u=\sigma^{-2}\Sigma \varphi^{T}t$$

Because $p(a,\sigma^{2}|t$)$\propto p\left(t|a,\sigma^{2}\right)p\left(a\right)p\left(\sigma^{2}\right)$ and $p(a,\sigma^{2}|t$) cannot be solved by means of integration, the solution is approximated using the maximum likelihood method, represented by

(17) $$\left(a_{MP},\sigma_{MP}^{2})=\underset{a,\sigma^{2}}{\arg}\;maxp(t|a,\sigma^{2}\right)$$

The iterative process of $a_{MP}$ and $\sigma_{MP}^{2}$ is given by:

(18) $$\{\begin{array}{c} a_{i}^{new}=\frac{\gamma_{i}}{\mu_{i}^{2}}\\ {\left(\sigma^{2}\right)}^{new}=\frac{{\left|\left|t-\varphi \mu \right|\right|}^{2}}{N-\sum_{i=0}^{N}\mu_{i}}\\ \gamma_{i}=1-a_{i}\sum^{\text{​}}i,i \end{array}$$

Here $\sum^{\text{​}}i,i$ is ith element in the Σ diagonal and the initial value of α and *σ*^2^ can be decided via the approximation of $a_{MP}$ and $\sigma_{MP}^{2}$ using Formula (15) continuously updated. After enough iterations, most of $a_{i}$ will be close to infinity, the corresponding parameters in $w_{i}$ will be zero, and other $a_{i}$ values will be close to finite. The resulting corresponding parameters $x_{i}$ of $a_{i}$ are now referred to as the relevance vector.

### 3.5. Performance Evaluation

In the paper, we used the following evaluation criteria as a measure for evaluating the performance of the proposed classifier and feature extraction method in our experiment. There are Ac (Accuracy), Sn (Sensitivity), Pe (precision), and Mcc (Matthews’s correlation coefficient). The definition is as follows:

(19) $$\mathrm{Ac}\;=\;\frac{TP+TN}{TP+FP+TN+FN}\;\mathrm{Sn}\;=\;\frac{TP}{TP+TN}\;\mathrm{Pe}\;=\;\frac{TP}{FP+TP}\;\mathrm{Mcc}\;=\;\frac{\left(TP\;\times \;TN\right)-\left(FP\;\times \;FN\right)}{\sqrt{\left(TP+FN\right)\;\times \;\left(TN+FP\right)\;\times \;\left(TP+FP\right)\;\times \;\left(TN+FN\right)}}$$

where true positives (*TP*) represents the number of positive pairs that are predicted as interacting drug–target pairs, false positives (*FP*) is the count of negative pairs that are predicted as interacting drug–target pairs, true negatives (*TN*) is the total of negative pairs that are predicted as non-interacting drug–target pairs and false negatives (*FN*) represents the number of positive pairs that are predicted as non-interacting drug–target pairs. In addition, the Receiver Operating Curve (ROC) was established to evaluate the performance of the proposed approach in the experiment.

## 4. Conclusions

In the paper, we proposed a novel computational approach based on protein sequence, namely PDTPS (Predicting Drug Targets with Protein Sequence), to predict drug–target interactions (DTI). The PDTPS method combines bi-gram probabilities (BIGP), Position Specific Scoring Matrix (PSSM), and Principal Component Analysis (PCA) with Relevance Vector Machine (RVM). In order to evaluate the prediction capacity of the PDTPS, we carried out the method on enzyme, ion channel, GPCR, and nuclear receptor datasets by using five-fold cross-validation tests. The proposed PDTPS method achieved average accuracy of 97.73%, 93.12%, 86.78%, and 87.78% on enzyme, ion channel, GPCR, and nuclear receptor datasets, respectively. The experimental results showed that our method has good prediction performance. Furthermore, in order to evaluate the prediction performance of the proposed PDTPS method, we compared it with the state-of-the-art support vector machine (SVM) classifier on enzyme and ion channel datasets and other existing methods on four datasets. The promising comparison results further demonstrate the efficiency and robustness of the proposed PDTPS method. This makes it a useful tool and suitable for predicting DTI, as well as performing other bioinformatics tasks. For future studies, more effective feature extraction approaches and machine learning algorithms can be developed for predicting DTI.

## Figures and Tables

**Table 1:** 5-fold cross validation results performed by proposed model on an enzyme dataset.

Testing Set	Ac (%)	Sn (%)	Pe (%)	Mcc (%)
1	97.95	98.31	97.65	95.98
2	97.52	95.84	99.31	95.16
3	97.26	97.29	97.29	94.68
4	98.29	98.44	98.10	96.64
5	97.61	97.34	97.69	95.33
Average	97.73 ± 0.40	97.44 ± 1.04	98.01 ± 0.78	95.56 ± 0.76

**Table 2:** 5-fold cross validation results performed by proposed model on an ion channel dataset.

Testing Set	Ac (%)	Sn (%)	Pe (%)	Mcc (%)
1	92.71	91.18	94.58	86.48
2	91.02	92.31	89.49	83.64
3	94.41	94.46	94.14	89.44
4	93.39	93.81	94.10	87.55
5	94.07	94.85	92.47	87.18
Average	93.12 ± 1.34	93.32 ± 1.54	92.96 ± 2.10	87.18 ± 2.28

**Table 3:** 5-fold cross validation results performed by proposed model on a GPCR dataset.

Testing Set	Ac (%)	Sn (%)	Pe (%)	Mcc (%)
1	83.07	77.88	83.02	71.21
2	88.58	86.86	91.54	79.70
3	87.41	85.40	90.70	77.91
4	85.83	86.15	86.15	75.66
5	88.98	88.14	88.14	80.28
Average	86.77 ± 2.41	84.89 ± 4.04	87.91 ± 3.47	76.97 ± 3.64

**Table 4:** 5-fold cross validation results performed by proposed model on a nuclear receptor dataset.

Testing Set	Ac (%)	Sn (%)	Pe (%)	Mcc (%)
1	83.33	73.68	93.33	71.79
2	88.89	100.0	80.00	80.00
3	91.67	100.0	86.96	84.05
4	86.11	100.0	76.19	75.59
5	88.89	89.47	89.47	80.19
Average	87.78 ± 3.17	92.63 ± 11.53	85.19 ± 6.70	78.32 ± 4.72

**Table 5:** 5-fold cross validation results performed by SVM and RVM classifiers on an enzyme dataset.

Testing Set	Ac (%)	Sn (%)	Pe (%)	Mcc (%)
RVM + PSSM + BIGP				
1	97.95	98.31	97.65	95.98
2	97.52	95.84	99.31	95.16
3	97.26	97.29	97.29	94.68
4	98.29	98.44	98.10	96.64
5	97.61	97.34	97.69	95.33
Average	97.73 ± 0.40	97.44 ± 1.04	98.01 ± 0.78	95.56 ± 0.76
SVM + PSSM + BIGP				
1	90.94	90.56	91.48	83.52
2	89.49	91.18	88.67	81.15
3	90.60	93.06	88.85	82.93
4	92.48	94.11	90.95	86.08
5	92.24	93.97	90.29	85.67
Average	91.15 ± 1.23	92.57 ± 1.62	90.05 ± 1.25	83.87 ± 2.03

**Table 6:** 5-fold cross validation results performed by SVM and RVM classifier on an ion channel dataset.

Testing Set	Ac (%)	Sn (%)	Pe (%)	Mcc (%)
RVM + PSSM + BIGP				
1	92.71	91.18	94.58	86.48
2	91.02	92.31	89.49	83.64
3	94.41	94.46	94.14	89.44
4	93.39	93.81	94.10	87.55
5	94.07	94.85	92.47	87.18
Average	93.12 ± 1.34	93.32 ± 1.54	92.96 ± 2.10	87.18 ± 2.28
SVM + PSSM+ BIGP				
1	86.78	84.31	89.58	77.03
2	88.47	90.56	86.33	79.59
3	86.10	89.62	83.28	76.03
4	88.45	86.07	92.36	79.51
5	89.02	91.91	85.32	80.39
Average	87.77 ± 1.26	88.49 ± 3.18	87.37 ± 3.59	78.51 ± 1.87

**Table 7:** Comparison of predicting performance between our method and other methods on four Datasets.

Dataset	Our Method	DBSI [26]	Yamanishi [27]	KBMF2K [28]	NetCMP [29]
Enzymes	0.9773	0.8075	0.821	0.832	0.8251
Icon Channels	0.9312	0.8029	0.692	0.799	0.8034
GPCRs	0.8677	0.8022	0.811	0.857	0.8235
Nuclear Receptors	0.8778	0.7578	0.814	0.824	0.8394
